# Measuring Gait Variables Using Computer Vision to Assess Mobility and Fall Risk in Older Adults With Dementia

**DOI:** 10.1109/JTEHM.2020.2998326

**Published:** 2020-05-28

**Authors:** Kimberley-Dale Ng, Sina Mehdizadeh, Andrea Iaboni, Avril Mansfield, Alastair Flint, Babak Taati

**Affiliations:** 1 Toronto Rehabilitation Institute (KITE)University Health Network 7989 TorontoONM5G 2A2Canada; 2 Institute of Biomaterials and Biomedical Engineering, University of Toronto 7938 TorontoONM5SCanada; 3 Department of PsychiatryUniversity of Toronto 7938 TorontoONM5SCanada; 4 Centre for Mental HealthUniversity Health Network 7989 TorontoONM5T 1L8Canada; 5 Sunnybrook Research Institute 282299 TorontoONM4N 3M5Canada; 6 Department of Physical TherapyUniversity of Toronto 7938 TorontoONM5SCanada; 7 Department of Computer ScienceUniversity of Toronto 7938 TorontoONM5SCanada

**Keywords:** Computer vision, dementia, gait, stability, falls, pose tracking

## Abstract

Fall risk is high for older adults with dementia. Gait impairment contributes to increased fall risk, and gait changes are common in people with dementia, although the reliable assessment of gait is challenging in this population. This study aimed to develop an automated approach to performing gait assessments based on gait data that is collected frequently and unobtrusively, and analysed using computer vision methods. Recent developments in computer vision have led to the availability of open source human pose estimation algorithms, which automatically estimate the joint locations of a person in an image. In this study, a pre-existing pose estimation model was applied to 1066 walking videos collected of 31 older adults with dementia as they walked naturally in a corridor on a specialized dementia unit over a two week period. Using the tracked pose information, gait features were extracted from video recordings of gait bouts and their association with clinical mobility assessment scores and future falls data was examined. A significant association was found between extracted gait features and a clinical mobility assessment and the number of future falls, providing concurrent and predictive validation of this approach.

## Graphical abstract

Using recorded videos of natural gait of people with dementia, we showed concurrent and predictive validation for the use of human pose estimation for gait assessment and eventual use for fall prevention.

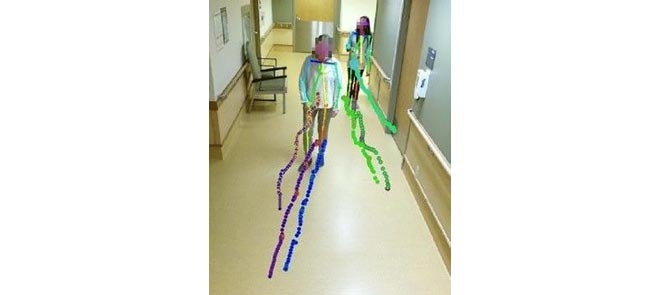

## I. Introduction

Dementia is a neurodegenerative disorder that impairs control of gait and increases the risk of falling [1]. In addition to being associated with a decrease in quality of life, loss of mobility and mortality, falls are also a large contributor to national healthcare costs [2]. While gait assessment is an important component of determining fall risk in older adults with dementia [1], current methods have important limitations, particularly for older adults with dementia.

Numerous technologies exist which can be used to reduce or eliminate the subjectivity of gait assessment. The majority of these tools, however, require expensive and difficult-to-use hardware, e.g. motion capture systems. Technologies that require sensors to be attached to the body or walking to be constrained are particularly difficult to implement for the dementia population.

In this work, we 1) apply recent advancements of human pose estimation into a system to process videos of older adults with dementia as they walk naturally in their place of residence, 2) extract several gait features from extracted pose sequences, and 3) examine the relationship between extracted gait features and clinical mobility scores (Performance Oriented Mobility Assessment (POMA)), as well as prospective fall events.

## II. Background and Approach

### A. Clinical Gait Assessment

Over the past two decades, functional assessments have been the most commonly used type of gait assessments for older adults [3]. These tools generally test the limitations of the patients’ gait and balance and require a trained clinician to score aspects of a patient’s gait based on observations. Due to the subjective nature of these test, results can vary based on the assessor [4]. In addition, they are performed infrequently due to lack of resources such as trained staff and specialized equipment [4], [5]. Gait and balance are two factors that contribute to fall risk, therefore functional assessments are often used as part of fall risk assessment. An example of a functional gait assessment is the Performance Oriented Mobility Assessment (POMA). POMA was first published as a functional mobility assessment specifically geared towards institutionalized older adults [6]. The test is split into two sections, POMA-gait and POMA-balance. Each section is comprised of specific tasks that the patient is prompted to do. The tasks for POMA-gait include step length and height, step symmetry, straightness of path, and sway of the trunk. The POMA-balance tasks include balance while standing, balance while sitting, ability to stand with eyes closed, and ability to turn 360 degrees [6]. The execution of each task is then assigned a score, and the task scores are added together to make the score for that section of test. For POMA, a high score indicates good mobility and balance. Inversely, low scores indicate poor gait and balance. Low scores can, therefore, indicate an increased fall risk.

POMA-balance and POMA-gait are reliable and valid for institutionalized cognitively intact older adults with interrater reliability scores of R = 0.80-0.95 and R = 0.72-0.86 [4], [7] for POMA-balance and gait, respectively. However for patients with dementia, functional assessments are difficult to perform due to communication barriers and lack of patient motivation [8]. Often, in such difficult cases, gait assessment is foregone due to a lack of available alternative.

Aside from the clinical use of POMA or other functional gait assessments, research has explored the correlations between quantitative gait variables and fall risk for older adults with dementia. Features of gait including gait speed or cadence, step length and width variability, and medio-lateral margin of stability have been associated with falls in older adults with dementia [9]–[11]. There are many studies which propose measuring these gait variables using devices such as inertial measurement units (IMUs) [12], mats with imbedded sensors, [13] or other wearable devices [14]. While some studies have been successful at assessing mobility in the dementia population with wearable sensors over a few days [15], other studies describe difficulties associated with sensor upkeep and acceptance in this population [16]. Thus, for longitudinal data collection over weeks and months, ambient collection has some important advantages over wearables.

### B. Human Pose Estimation

Human Pose estimation is the ability of a computer to determine the posture and limb articulation of a person from an image or video [17]. This has historically been a difficult computer vision problem due to the extensive number of variables present in pose estimation including variation not only in the subjects (people) but also the ways in which they move [17]. In recent years, with the advances in deep learning algorithms, however, performance of human pose estimation algorithms has improved significantly [18]–[20].

Many of the recent publications aim to reduce the complexity of the network architecture and increase analysis speed and/or accuracy [18], [20], [21]. As these models have become more precise and require less computing power, they have also become viable for many applications, including clinical applications, where human movement analysis is the subject of interest. While 3D human pose estimation models have been developed and published [22]–[24], 2D human pose estimation models are more accessible both in availability of code and support as well as being able to process large amounts of video locally. Local processing (as opposed to cloud computing) was required due to the identifiable nature of the videos collected.

Thus far human pose tracking has been explored for ivarious methods of measuring gait and mobility in the older adult population, including automatically acquiring the clinical parameters measured in the timed up-and-go (TUG) test, which is a clinical measure of mobility [25], step monitoring [26], and general gait parameter extraction [27]. Another application which has been extensively researched is the detection of falls [28]–[30]. While many studies have used pose tracking to generally analyse gait or identify when a fall occurs, there is limited research addressing ways to prospectively determine when future falls are likely.

### C. Human Pose Estimation and Gait Assessment

Previous studies which address mobility assessment of older adults in assisted living homes have made use of human pose estimation to classify of movement type such as if the person is standing, sitting, or lying [31]. Other studies have used specialized depth sensors such as the Microsoft Kinect v2 sensor and its skeleton tracking capabilities to measure spatiotemporal and kinematic variables of gait [32]–[34]. For example, one study explored the use of Kinect for ongoing mobility monitoring of older adults after hip replacement surgery and confirmed the sensitivity of measured gait parameters during the course of recovery [35].

Recently, we established the feasibility of using a Kinect v2 sensor, in combination with radio-frequency identification (RFID), in a dementia residential setting to longitudinally collect parameters of gait as residents walk naturally in their environment [36]. We further investigated the association between baseline gait parameters, calculated from Kinect v2 skeletal tracking, and observational performance-based tests of gait and balance, as well as the number of future falls [11]. While the feasibility of the system was established, these studies also revealed some limitations of using the Kinect sensor for ambient monitoring. For instance, for a variety of reasons, in approximately half of the recorded walking bouts, skeletal tracking was not successful [36]. In addition, the Kinect depth of field is small, limiting the number of steps that could be captured in a walking bout. In the above studies, an average of 3.9 seconds of walking data per walking bout was usable for gait assessment [36]. If a regular camera could be used to perform human pose estimation and skeletal tracking, then a larger number of steps could potentially be analysed. It is also desirable to perform ambient vision-based monitoring using regular cameras, as opposed to special hardware (e.g. Kinect) to avoid the risk of obsolescence.

In this work, we explore the utility of vision-based human pose estimation, operating on regular image/video data and without the need for special sensors, for longitudinal gait monitoring in older adults with dementia. We compute a set of gait features based on a sequence of tracked poses, and examine the relationship between baseline parameters of gait computed this way and future incidents of falls and clinical scores of balance and mobility.

## III. Methods and Procedures

### A. Data Collection

Data were collected from 31 participating inpatients in the Specialized Dementia unit at the Toronto Rehabilitation Institute. All patients admitted to the unit are diagnosed with dementia. Those who were independently mobile were recruited to the study. Patients who used walking aids, such as walkers, were included in this study; but those with walking aids which occlude the patient’s legs, such as rollators, were excluded. (Rollators are used similarly to walkers but have additional wheels on the front legs and also a seat and sometimes a basket). The study protocol was approved by the institute’s Research Ethics Board. Written informed consent to the study was provided by substitute decision-makers, and each participant was only assessed or engaged in data collection if they gave verbal assent.

Color videos of $1920\times1080$ pixel resolution were collected at 30 frames per second, using an existing Microsoft Kinect v2 sensor which had been previously mounted on the ceiling at the end of a hallway of the inpatient dementia unit. The Kinect sensor incorporates an RGB (i.e. colour) camera and a depth sensor; for this study only the RGB videos were analysed. A Radio-Frequency Identification (RFID) system was used to identify patients and initiate recordings. Two polarized ultra-high frequency antennas were placed on the walls on each side of the hallway, 8 m from the camera. The antennas were camouflaged to detract attention from patients on the unit by covering the antennas with plywood which matched the finishing of the section of the wall to which they were attached. RFID tags of unique identifiers per participant were ironed inside each leg of each pair of trousers of the participants at the height of the antennas using a heat press. When a participant passed by the antennas, 30 seconds of video, capturing their walk along the hallway was automatically recorded.

Videos were collected from the time of each participant’s enrollment until either their discharge from the unit or the point at which they no longer met study criteria, e.g. were no longer able to independently walk 20 m. To increase the number of video recordings, a clinical research assistant encouraged participants to walk by the recording system daily. In total, 1066 standard videos were recorded. In addition to video collection, POMA-gait and POMA-balance scores were assessed upon enrollment to the study, and falls incidence were recorded throughout the study. For this study, falls were documented based on chart review, incident reports and discussion with clinical staff at daily safety huddles. Any incident where a participant unintentionally came to rest on the floor or any lower level was recorded as a fall.

### B. Application of Human Pose Estimation

**FIGURE 1.**
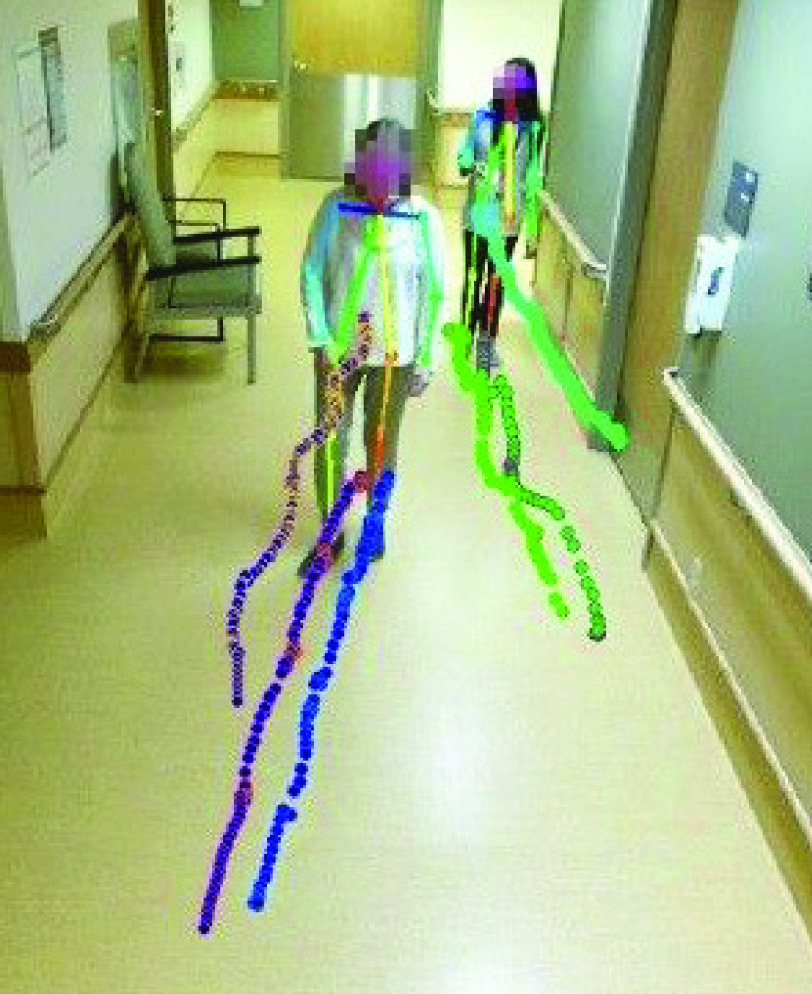
Ankle and eCOM keypoints for the participant and research assistant of a walking bout overlaid on the first frame of the video of that walk.

In visual preprocessing, approximately 20 % of the recorded videos, in which participants changed course (e.g. walked by the antenna to trigger video recording, but then turned and walked back), or in which participants did not walk independently (e.g. held the handrail), were discarded. OpenPose, a pre-trained deep learning-based human pose estimation model was then used to track 13 keypoints of interest over the remaining videos [20], [37]. In visual post-processing, any keypoints tracked from individuals other than the study participant, such as the clinical research assistant accompanying the patient, were discarded (e.g. Figure 1).

### C. Verification of Human Pose Estimation

**FIGURE 2.**
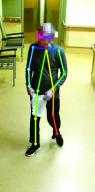
Sample image from a participant’s recorded walk, with the 13 detected keypoints of interest plotted on the image.

In order to verify the accuracy of OpenPose keypoint placement on the collected videos, a standard Percent Correct Keypoint analysis was performed [38]. First, two random frames of one video per participant were selected. Then, the 13 keypoints of interest (see Figure 2) were blindly hand annotated on each of the selected images. The output keypoints from OpenPose were then compared against the manually annotated keypoints and the Percentage of Correct Keypoints (PCK) was calculated. In the PCK analysis, the threshold was set to half the length of the head segment (PCK@0.5) [38]. The PCK@0.5 value was calculated three times: over all 13 keypoints over the two ankle keypoints, and over the two hip keypoints. A comparison between the correctness (i.e., placement of keypoints within the threshold) and the confidence level assigned to the keypoint placement was also made.

**FIGURE 3.**
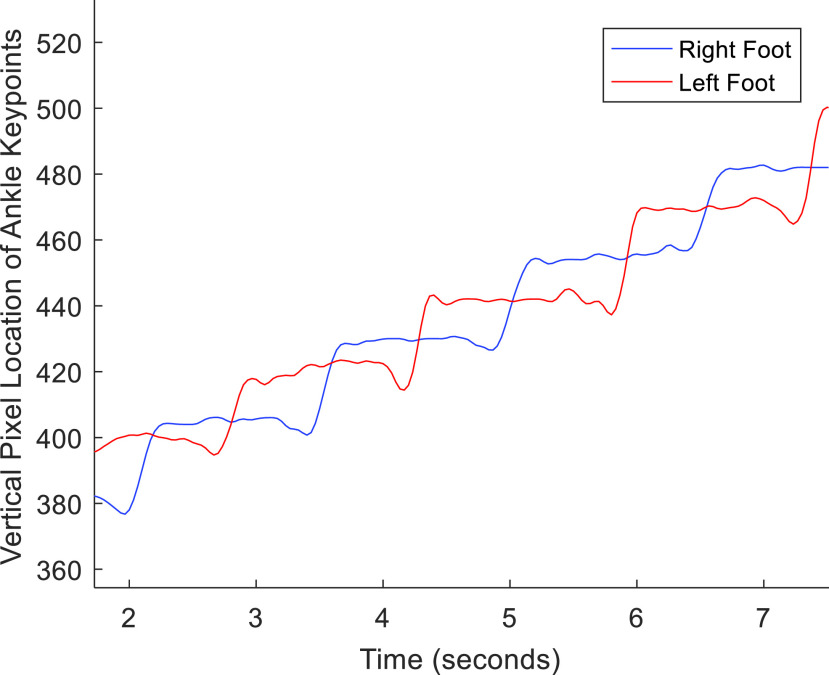
Vertical movement of ankle keypoints over time.

### D. Estimation of Gait Variables From Keypoints

In further post-processing, using Matlab (Mathwork Inc., Natick, USA), the time series of keypoints’ coordinates were first filtered using a second order lowpass butterworth filter with a cutoff frequency of 4 Hz. This cut-off frequency was determined through residual analysis [39]. The movements of keypoints frame to frame were then used to estimate gait variables for each recorded walk. In total, 7 gait variables were extracted from each walking sequence as described below.

Since tracked points are in 2D (pixel) coordinates, it was not possible to calculate gait variables requiring 3D distances, e.g. step length or velocity. However, temporal gait variables (e.g. cadence or step time) and relative distance measures (e.g. left/right symmetry of step width) could be measured. All pixel distances were normalized to the mediolateral hip span, i.e. the pixel distance between the left and right hip keypoints.

**FIGURE 4.**
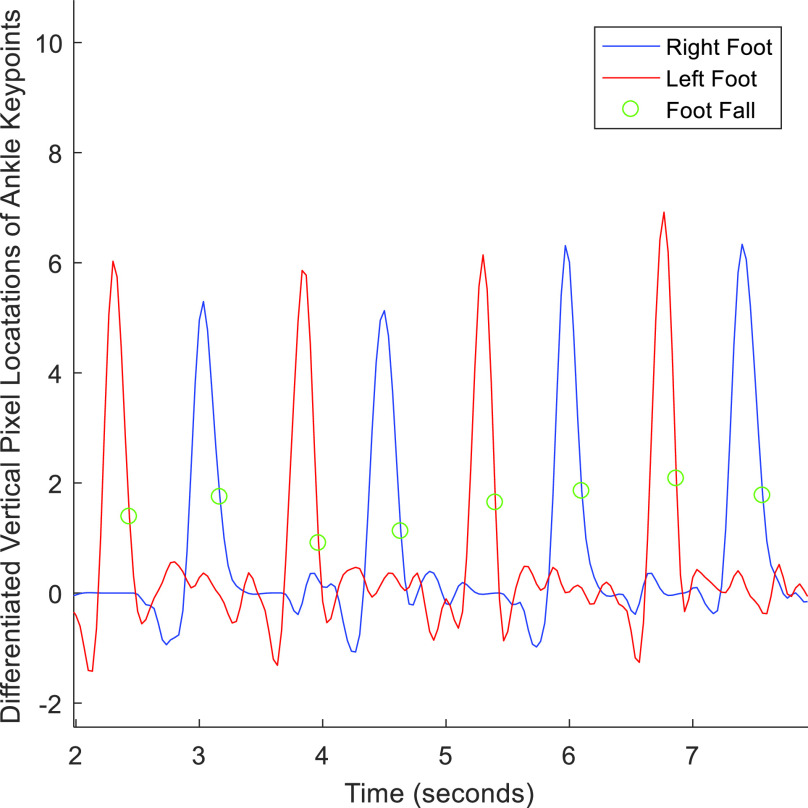
Foot strike analysis performed by differentiating ankle keypoint movement and finding 35% of maximum differential in speed [41].

To estimate foot strikes, the foot pixel velocity was first calculated by differentiating the vertical position of each ankle keypoint over time (see Figure 4). As suggested in [40] the foot strike was located where the signal velocity passed over the 35% threshold of the maximum differential of each step (see Figure 4). *Cadence* (i.e. number of steps per minute) was then calculated by finding the number of foot strikes divided by the total time of the walking bout in minutes. The *symmetry index (SI) of step times* was calculated by taking the absolute value of the difference of time spent on each foot, divided by half of the total time as in [41]. The *coefficient of variance (CV) of the step time* was calculated as the standard deviation of the time of each step divided by the average step time.

**FIGURE 5.**
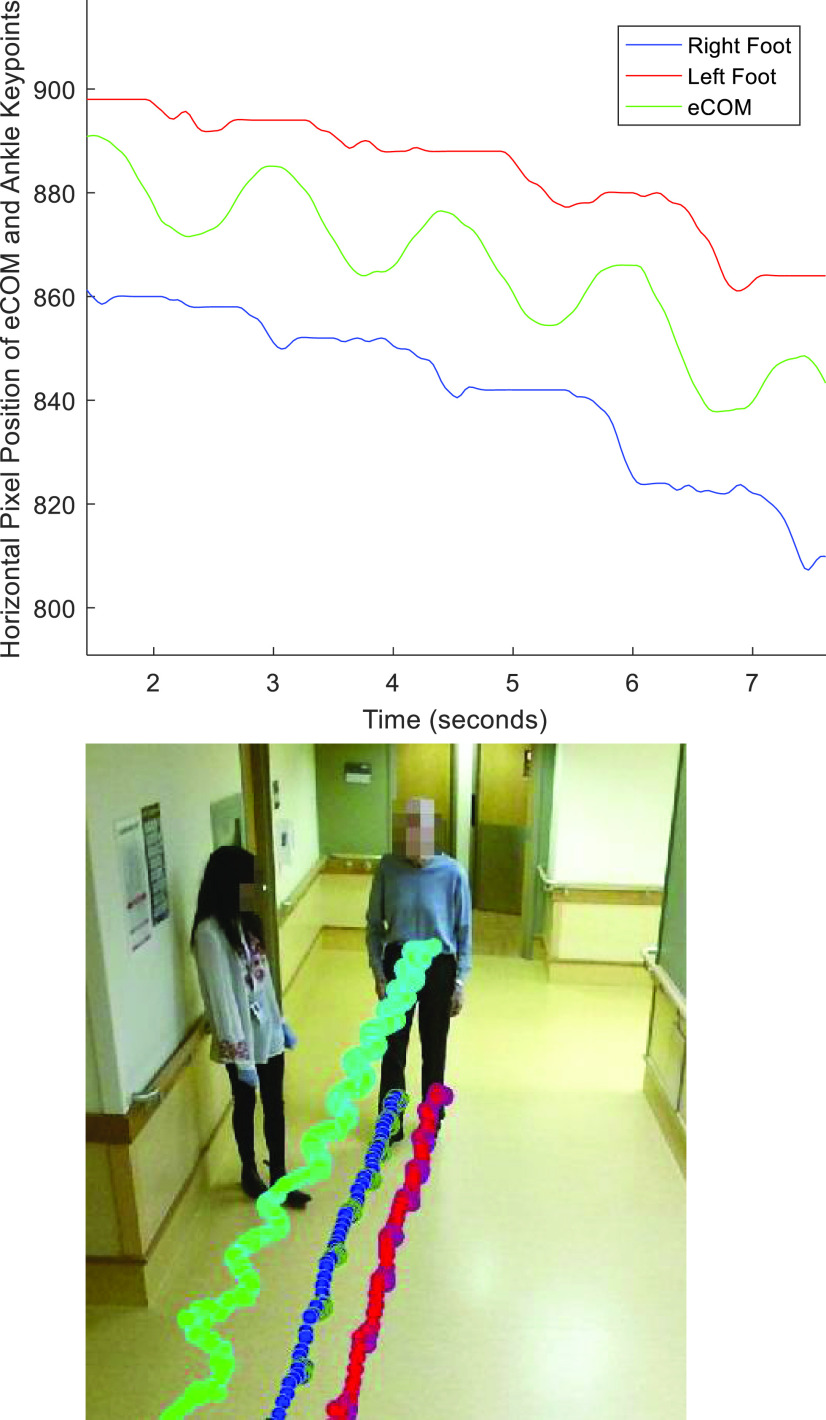
Horizontal movement of the ankle keypoints and eCOM over time for one walking bout, (top) and corresponding keypoints plotted on the image of the first frame of the video (bottom).

Average step width was calculated as the average of the pixel distances between ankle keypoints, divided by the pixel distances between hips per frame (see Figure 5). The CV of step width was calculated in the same fashion as CV of step times. Two additional gait variables were calculated estimating a measure of margin of stability (MOS) [42]. To do so, the estimated centre of mass (eCOM) was first determined as the centre point between the two hip keypoints. The estimated extrapolated centre of mass (eXCOM) was then calculated by adding to eCOM its normalized pixel velocity divided by the square root of gravity divided by leg length [43]. Leg length in this instance was determined as the normalized pixel length between the hip keypoint and ankle keypoint at heel strikes, averaged over all heel strikes, and then averaged between left and right legs. For each frame, the horizontal pixel difference from the eXCOM to the right and left ankle keypoint of the stance foot was calculated and normalized, and this measure was averaged over each frame of the walk to get the average estimated margin of stability (eMOS) variable [11]. The minimum eMOS was calculated similarly, but by averaging only the smallest eMOS measure per step of the walk [11].

These estimated gait variables were calculated for each video of each participant and averaged over the first two weeks of their enrollment in the study comprising participant’s “baseline gait variables”.

### E. Implementation Details

A Tensorflow implementation of the two dimensional human pose estimation algorithm, OpenPose [20], [37], [44] was used to place the keypoints used in this analysis. The model used was pre-trained on the Max Planck Institut Informatik (MPII) Dataset [38]. The camera used in data collection was a Microsoft Kinect for Windows v2, but only the recorded RGB streams were used in this analysis ($1080\times 1920$ pixel resolution, 30 frames per second). Calculation of the gait features and statistical analyses were performed using Matlab (Mathwork Inc., Natick, USA).

### F. Outcome Measure

The outcome measures were 1) POMA-gait sub-scale score and POMA-balance sub-scale score and 2) the number of falls that each participant experienced while participating in the study. Falls were recorded prospectively by the clinical research assistant who gathered details directly from unit staff members and continuously reviewed falls documentation in charts.

### G. Statistical Analysis

Univariate linear regression analysis was performed to determine the correlation between the POMA-gait scores of the participants with their estimated gait variables. This analysis was repeated between the POMA-balance scores and estimated gait variables. Similarly, univariate Poisson regression analysis was performed to determine the correlation between each of the estimated gait variables and the number of falls recorded during the study for each participant. The length of participation in the study was included in the Poisson regression as an exposure (offset) variable. Statistical significance was set to $p < 0.05$ for all three regression analyses. Finally, the gait variables which were found to have statistically significant association to the number of falls were then used to make a multivariate model, correlating the estimated gait variables to number of falls.

## IV. Results

### A. Participants and Video Data

Between November 2017 to December 2018, 33 participants were recruited to the study. One participant withdrew consent and one was found not to meet the inclusion criteria, leaving a total of 31 participants for analysis. In total, 1066 colour videos (i.e. walking bouts) were collected of the remaining 31 participants during their respective frist two weeks in the study. In post processing, 19.7% of recorded videos were discarded and not analysed due to any of the following factors: the participant holding the handrails while walking, the participant turning around to walk away from the camera, participants completing less than 3 steps in the recording time, or the participant being significantly occulded from the view of the camera. The average number of videos analysed per participant was 33 ± 24, with 6 being the lowest number of videos and 103 being the largest. The average length of inclusion in the study (during which falls were recorded) was 44 ± 19 days, with an average of 1.9 ± 1.1 walks per day per participant. The average time of recording of each walk was 4.7 seconds. The characteristics of the participants, separated into fallers and non-fallers, are summarized in Table 1.

**TABLE 1:** Participant Characteristics

	Non-Fallers	Fallers
Age	75.5 ± 8.6	78.4 ± 89
Sex (% female)	36	39
POMA-balance score	9.8 ± 2.9	8.0 ± 3.4
POMA-gait score	8.7 ± 2.1	7.6 ± 3.0
Number of walks recorded	65.7 ± 43.6	73.2 ± 71.7
Number of falls recorded	0	2.5 ± 1.7
Duration in the study (days)	40.3 ± 12.6	72.7 ± 44.2

### B. Verification of Human Pose Estimation

A PCKh@0.5 score of 87% was obtained when comparing OpenPose keypoints to blindly hand annotated keypoints. The PCKh@0.5 value for the hips and the ankles were 79% and 84% respectively. OpenPose outputs a confidence rating of each keypoint ranging from 0 (no confidence) to 1 (full confidence). Using these confidence scores, keypoints detected with low confidence could be discarded, for example to be imputed using the corresponding keypoints from temporally adjacent frames. Table 2 reports the PCKh@0.5 values for all 13 keypoints and for the two ankle keypoints achieved after discarding keypoints with confidence scores below 0.2, 0.4, 0.6 or 0.8. It can be seen in table 2 that at a cutoff threshold of 0.4, less than 20% of keypoints are discarded, and the all keypoints achieve a PCKh@0.5 score of 90%. While higher thresholds result in higher PCKh@0.5 scores, they also result in a high percentage of keypoints being discarded. Therefore, the confidence score threshold of 0.4 was selected as an appropriate trade off, and keypoints detected with confidence scores below 0.4 were discarded and imputed (linearly) based on coordinates from temporally adjacent frames.

**TABLE 2:** PCKh@0.5 for All Keypoints and for the Two Ankle Keypoints With Cut Off Thresholds of Confidence Scores at 0.2, 0.4, 0.6, and 0.8

	PCKh@0.5		
Threshold	All keypoints	Ankles	% of all keypoints discarded per threshold
0.2	88.9	84.1	7.1
0.4	91.3	89.8	18.5
0.6	90.9	93.2	36.7
0.8	93.5	100.0	76.2

### C. Regression to POMA Scores

The average POMA-gait score of the 31 participants analysed was 8.2 ± 2.6 with a range of 4–12 while the average POMA-balance score was 8.8 ± 3.4 with a range of 2-14.

Table 3 shows the results of univariate linear regression from each of the estimated gait variables to POMA-gait scores. Only cadence was found to be significantly associated with POMA-gait score (p = 0.00047).

**TABLE 3:** Linear Regression to POMA-Gait

Gait Variable	Regression Coefficient	SE	t	p	R^2^
Cadence (steps per minute)	−0.15	0.038	−3.9	0.00047	0.35
Average eMOS	−2.3	5.6	−0.41	0.68	0.01
Minimum eMOS	−5.3	4.5	−1.2	0.24	0.05
Average step width	1.4	2.6	0.54	0.59	0.01
Average step width	−0.19	0.74	−0.25	0.80	0.00
CV step time	1.00	4.6	0.22	0.83	0.00
SI step time	0.038	0.035	1.1	0.28	0.04

Table 4 shows the results of univariate linear regression from each of the estimated gait variables to POMA-balance scores. Cadence and average step width were found to be significantly associated with POMA-balance (p < 0.05).

**TABLE 4:** Linear Regression to POMA-Balance

Gait Variable	Regression Coefficient	SE	t	p	R^2^
Cadence (steps per minute)	−0.15	0.05	−2.82	0.0085	0.22
Average eMOS	9.6	7.02	1.37	0.18	0.061
Minimum eMOS	2.3	5.91	0.39	0.70	0.0051
Average step width	7.3	3.05	2.38	0.024	0.16
CV step width	−0.55	0.95	−0.58	0.57	0.010
CV step time	−4.34	5.87	−0.74	0.47	0.019
SI step time	0.00	0.05	0.04	0.97	0.000042

### D. Poisson Regression to Number of Falls

Of the 31 participants whose data was used for this analysis, there were 17 fallers and 14 non-fallers. For the fallers, the average number of falls was 2.6 ± 1.7, with a range of 1 to 8 falls. Table 5 shows the results of the univariate Poisson regression to the number of falls.

**TABLE 5:** Univariate Poisson Regression to Number of Falls

Gait Variable	Regression Coefficient	Odds Ratio	SE	t	p	R^2^
Cadence (steps per minute)	0.05	1.06	0.014	3.9	0.00010	0.21
Average eMOS	−8.3	0.00025	2.3	−3.7	0.00024	0.25
Minimum eMOS	−3.2	0.041	1.3	−2.5	0.012	0.086
Average step width	−3.3	0.037	0.91	−3.6	0.00031	0.24
CV step width	−0.074	0.93	0.27	−0.28	0.78	0.031
CV step time	2.0	7.4	1.3	1.5	0.12	0.12
SI step time	−0.0065	0.99	0.011	−0.59	0.56	0.035

It was found that cadence, average eMOS, minimum eMOS, and average step width were significantly correlated to the number of falls experienced ($p < 0.05$). For the multivariate analysis, the significantly associated ($p < 0.05$) gait variables were selected. Of the gait variables that were highly correlated, i.e., the two measures of eMOS, the gait variable of strongest association, determined by highest R^2^ value, was selected. This resulted in a multivariate model with an R^2^value of 0.42, and an R^2^value adjusted for the number of predictors of 0.59 with cadence and average eMOS retaining significant association, as seen in Table 6.

**TABLE 6:** Multivariate Poisson Regression, ($\text{R}^{2}=0.42$, Adjusted $\text{R}^{2}=0.59$)

Gait Variable	Regression Coefficient	SE	t	p
Cadence (steps per minute)	0.064	0.016	3.9	0.000087
Average eMOS	−11.5	3.9	−2.9	0.0033
Average step width	0.77	1.5	0.51	0.61

Cadence was the only extracted gait variable found to be associated to the POMA-gait scores. This may in part be attributed to the previously reported limitations in validity of the POMA-gait specifically for the dementia population [3], [11]. The POMA-balance scores were associated with cadence and average step width. Participants with higher cadence (more steps per minute) had worse POMA-balance scores, which can, clinically, contribute to an increase in fall risk. This finding was different to previous findings in older adults, where a decrease in cadence was associated with increased fall risk [32], [33]. However, in the dementia population, an increase in cadence was associated with movement disorders such as parkinsonism. Another possibility was that increased cadence in those with dementia could represent a failure to self-regulate in response to poor balance and therefore increased fall risk [33]. Step width was also associated with POMA-balance, with a wider step width associated with increased POMA-balance scores, indicating better balance and therefore a decreased fall risk. This aligns with expectations as a wider step width means an increased base of support and therefore more stability.

Gait variables calculated from pose-tracking were also predictive of the participants’ number of future falls which occurred over the length of the study. Similar to the association with POMA-balance, an increased cadence was associated with an increase in number of falls, and a wider step width was associated with a decrease in number of falls.

The eMOS measures were also found to be associated to number of falls. Lower average and minimum eMOS, were associated with increased number of falls. A model including cadence, average eMOS, and average step width was able to explain 59% of the variance in number of falls experienced by the participants. For this multivariate model, the minimum eMOS was excluded as it was highly correlated to the average eMOS variable. The associations found show promise for future work to develop a predictive model using extracted gait variables to predict future falls.

## V. Discussion

In this study, we demonstrated that human pose estimation can be used to extract clinically meaningful data from video collected of older adults with dementia walking in environment in which they live. This method improves upon existing clinical gait assessment methods as we are able to analyse natural walking in a residential environment, and do so over time. Importantly, this study supports an ambient data acquisition method without the need to attach any sensors to the participants, which significantly reduces the burden of implementation to clinical staff compared to methods which require continual upkeep of wearables. The use of wearable sensors in the dementia population is particularly challenging because of non-adherence. By contrast, ambient monitoring systems are low effort on the part of patients and clinical staff and are thus well suited to this population and environment. Compared to a previous study which extracted gait variables from videos recorded with Kinect [11], [36], our system was able to capture videos of walking bouts which were 20% longer per recording, on average. The ability to analyze longer walking bouts, and therefore more steps per bout, increases the accuracy of the gait variable extraction by allowing for better normalization of the per step variables extracted from each walking bout. Additionally, this study used open source algorithms to perform the human pose estimation used for analysis, which allowed increased automation of the data extraction and analysis. Furthermore, pose estimation in video allows for the use of regular cameras, as opposed to special hardware (e.g. Kinect). Video cameras are ubiquitous and are already present in many long-term care facilities in the form of security cameras. This study showed that gait parameters calculated from video cameras has clinical utility. Specifically, extracted gait variables were shown to be clinically meaningful by the associations determined through a series of regressions to POMA scores and observed instances of future falls.

### A. Limitations

Some difficulties were encountered during the data collection and analysis for this study. The majority of these challenges were due to the lack of infrastructure existing in clinics to store large volumes of data paired with the cautious nature of healthcare institutions when it comes to collecting and storing video data. Privacy protocols required all videos to be stored on hospital servers, which meant having to deal with high traffic and therefore slow data transfers. Additionally, the use of a computer cluster for processing was not possible due to the inability to de-identify the videos, so the human pose estimation was performed locally on a single graphics processing unit (GPU), an Nvidia GTX 1080 which increased the computation cost.

One limitation of this study is the limited time of participation in the study. Due to the nature of the specialized dementia unit, patients are typically admitted for 6–8 weeks, limiting data collection to that time, and limiting the ability to perform longer term longitudinal analysis. An additional limitation is that documentation of falls did not differentiate between types of falls (e.g. from standing, from sitting, from the bed).

We also note that, with the exception of temporal measures, the gait measures calculated in this study are not comparable to true spatial gait variables. The gait variables measured in this study were relative measures and unitless, e.g. step width normalized to the mediolateral hip span. While these unitless measures were based on clinical measures of gait, they could not be used to compare directly to spatial gait variables measured by traditional means, e.g. force plates or optical motion capture. Nevertheless, we note that the main utility of gait assessment is the relationship between assessed gait and clinical scores or clinically relevant events (e.g. falls). Gait parameters calculated in this study showed associations with clinical measures (concurrent validity) and with future falls (predictive validity) and, as such, may still be useful for predicting which patients are at risk of falling even though they do not directly correspond with gait parameters measured in the laboratory.

### B. Future Work

This study provides the foundation for future longitudinal studies using video to assess gait quality in natural walking.

Once more longitudinal data becomes available, a transition from regression analyses to predictive modelling should be explored. If validated as an effective method of predicting short term fall risk, this method could be implemented as a real time gait monitoring and assessment tool. This tool could function as a warning system, pushing alerts to care staff to intervene when a patient’s gait indicates high risk of falls.

Machine learning should also be explored as a refinement to this method. Machine learning could lead to determining stronger associations than statistical regression analyses. Machine learning could also be used to analyse the raw human pose estimation data rather than first extracting gait variables from the human pose estimation data, then drawing conclusions directly from the human pose estimation data and therefore streamlining the process.

An additional future research question which could be explored is to concurrently measure gait parameters with the methods from this study as well as with traditional motion capture systems to compare the relative (normalized) gait measures to their absolute (i.e. not normalized) counterparts. While this study showed associations directly between clinical outcome measures and the relative gait measures, should this method be validated by comparison to absolute gait measures, it may be possible to expand the use of these methods to other applications which require absolute gait measures. When comparing to falls incidence, future studies should compare the measured gait features only to falls which occurred due to loss of balance during walking rather than all falls.

Another way to expand the scope of this study in the future would be to move from 2D to 3D human pose estimation algorithms. Numerous papers and algorithms have been published, suggesting the ability to infer a 3D pose from image or video input [22]–[24]. Should these algorithms be verified as precise on our dataset, then the gait variable extraction could be expanded to include variables measures not only along the mediolateral axis, but also along the anterior-posterior axis.

## VI. Conclusion

Using recorded videos of natural gait of people with dementia, we showed concurrent and predictive validation for the use of human pose estimation for gait assessment. Gait variables extracted from the human pose estimation data are associated with both clinical gait measures and with future observed falls. This work sets the stage for future validation studies, and for the development of real-time gait monitoring and assessment as well as the development of a fall prediction systems.
